# Supplementary material for: Are International Units of Anti-HBs Antibodies Always Indicative of Hepatitis B Virus Neutralizing Activity?
Source: Vaccines (Basel). 2023 Apr 4;11(4):791. doi: 10.3390/vaccines11040791 (PMC10147002; doi:10.3390/vaccines11040791)
Supplement: Supplementary file 1 [file vaccines-11-00791-s001.zip › vaccines-2295749-Supplementary.pdf]

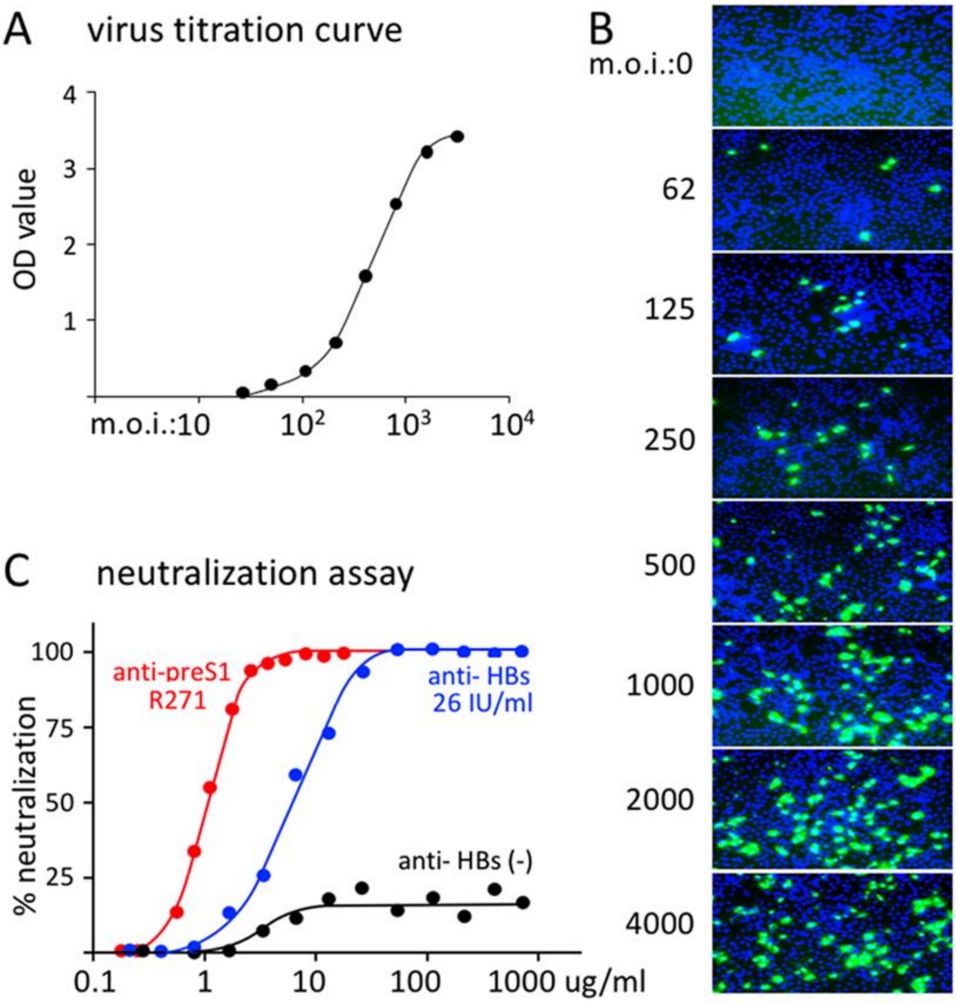

Figure S1: In vitro neutralization assay. Response of Huh-106 cells to infection with HDV measured by ELISA quantification of intracellular HDAg at 9 dpi (A), or by fluorescent antibody staining (B) indicates that response to infection is dose dependent for 100<m.o.i<1000. Neutralization of infection with polyclonal anti-HBs, or anti-preS1, antibodies is dose dependent.

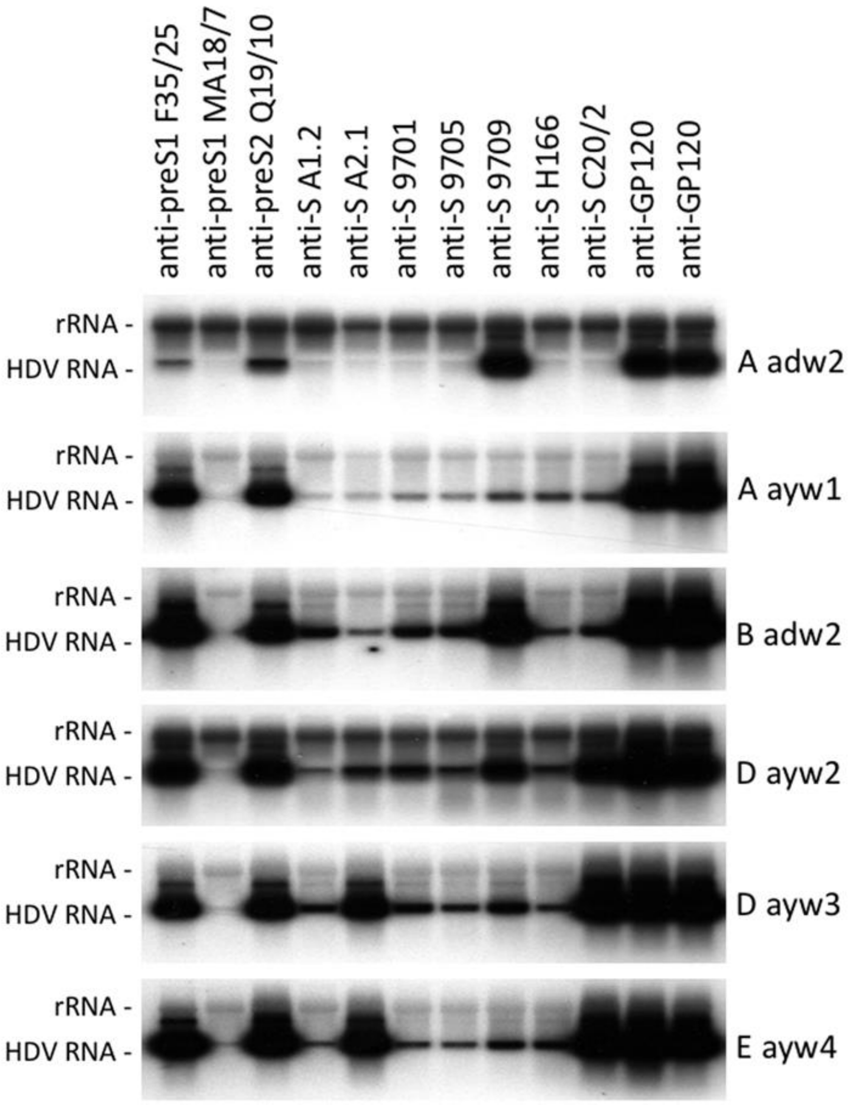

Figure S2: Monoclonal anti-HBs antibodies directed to the HBsAg "a" determinant. neutralize HDV infectivity according to HBV envelope protein serotypes. Huh106 cells were inoculated with HDV virions bearing the envelope proteins of HBV genotype Aadw2, A-ayw1, B-adw2, D-ayw2, D-ayw3 and E-ayw4 in the presence of anti-preS1 mAbs F3525 and MA18/7, anti-preS2 Q19/1, anti-HBsAg mAbs A1.2, A2.1, 9701, 9705, 9709, H166, C202 and anti-HIV GP120 at a 0.05 µg/mL concentration. Infection was assessed by measuring the accumulation of intracellular HDV RNA at 9 dpi. rRNA, ribosomal RNA.

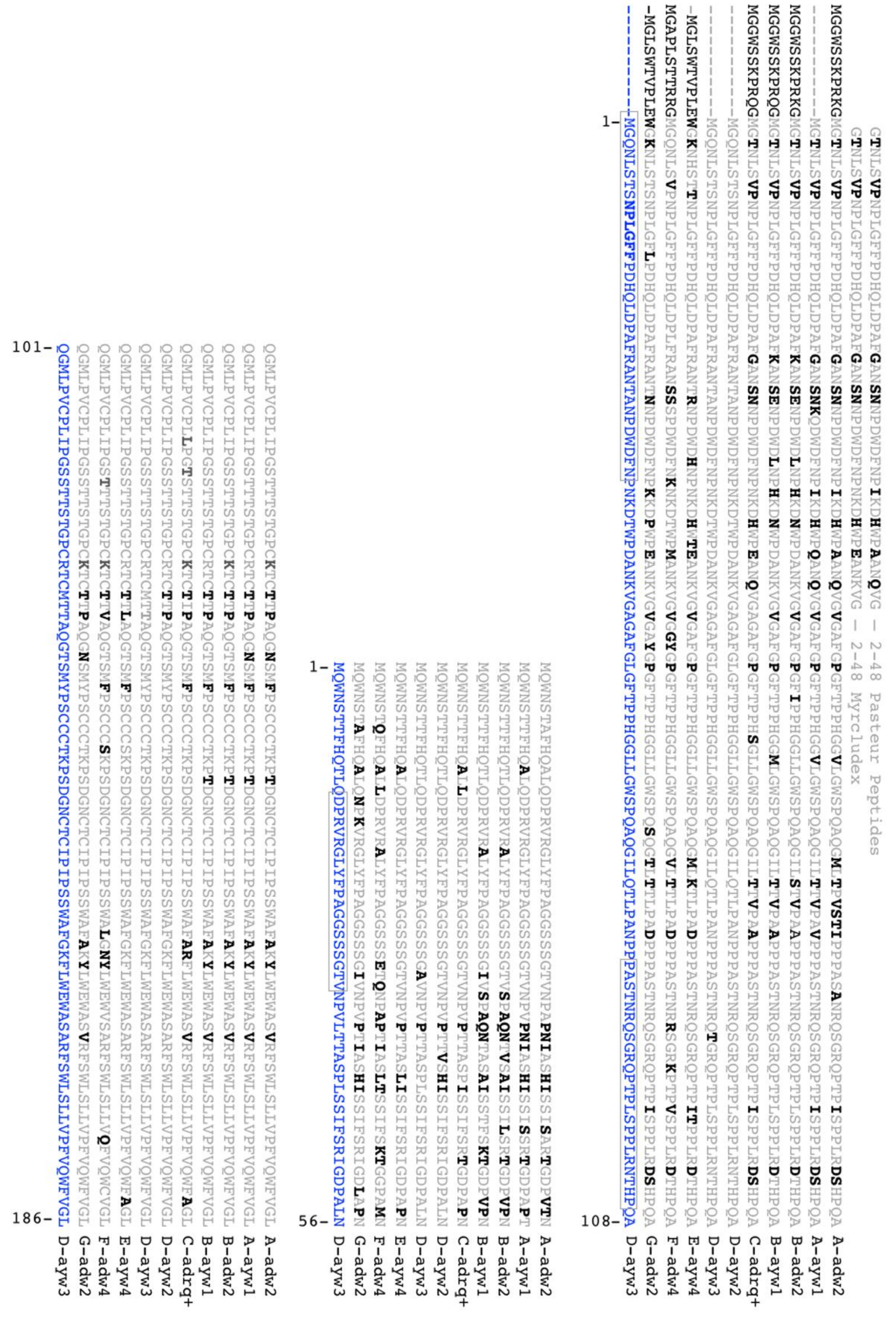

Figure S3: Amino acid sequence comparison of the HBV envelope protein domains pre-S1(aas 1-108, pre-S2 (aas 1-56)and AGL(aas 101-186) of genotype A-subtype adw2 (Genebank: MW357582), A-ayw1(Genebank: MW357583), B-adw2 (Genebank: MW357584), B-ayw1 (Genebank: MW357585), C-adrq+ (Genebank: MW357586), D-ayw2 (Genebank: MW357587), D-ayw3 (Genebank: MW357588), Eayw4 (Genebank: MW357589), F-adw4 (Genebank: MW357590), G-adw2 (Genebank: MW357591) and laboratory reference D-ayw3 (Genebank: X85254). Bold letters indicate amino acids change in comparison to the laboratory sequence (D-ayw3).
